# Supplementary material for: Modeling glioblastoma heterogeneity as a dynamic network of cell states
Source: Mol Syst Biol. 2021 Sep 16;17(9):e10105. doi: 10.15252/msb.202010105 (PMC8444284; doi:10.15252/msb.202010105)
Supplement: Supplementary file 6 — Source Data for Figure 5 [file MSB-17-e10105-s004.zip › Figure5A_sourcedata/GSEA_3071/hallmarks_stateII.GseaPreranked.1623742536692/HALLMARK_APOPTOSIS.html]

Details for gene set HALLMARK\_APOPTOSIS[GSEA]

|  || Dataset | state23071 |
| Phenotype | NoPhenotypeAvailable |
| Upregulated in class | na\_neg |
| GeneSet | HALLMARK\_APOPTOSIS |
| Enrichment Score (ES) | -0.2619245 |
| Normalized Enrichment Score (NES) | -1.2782031 |
| Nominal p-value | 0.16784452 |
| FDR q-value | 0.41655356 |
| FWER p-Value | 0.98 |
Table: GSEA Results Summary

  

Fig 1: Enrichment plot: HALLMARK\_APOPTOSIS      
 Profile of the Running ES Score & Positions of GeneSet Members on the Rank Ordered List

  

| PROBE | GENE SYMBOL | GENE\_TITLE | RANK IN GENE LIST | RANK METRIC SCORE | RUNNING ES | CORE ENRICHMENT || 1 | BRCA1 |  |  | 49 | 1.249 | 0.0247 | No |
| 2 | TOP2A |  |  | 72 | 1.132 | 0.0526 | No |
| 3 | CAV1 |  |  | 134 | 0.988 | 0.0666 | No |
| 4 | GADD45B |  |  | 146 | 0.965 | 0.0923 | No |
| 5 | HMGB2 |  |  | 276 | 0.780 | 0.0833 | No |
| 6 | EBP |  |  | 336 | 0.721 | 0.0899 | No |
| 7 | PMAIP1 |  |  | 426 | 0.666 | 0.0875 | No |
| 8 | IL18 |  |  | 427 | 0.666 | 0.1071 | No |
| 9 | CDK2 |  |  | 530 | 0.612 | 0.0999 | No |
| 10 | BTG3 |  |  | 540 | 0.607 | 0.1156 | No |
| 11 | TNFRSF12A |  |  | 629 | 0.566 | 0.1105 | No |
| 12 | WEE1 |  |  | 685 | 0.536 | 0.1126 | No |
| 13 | BCL2L1 |  |  | 687 | 0.536 | 0.1282 | No |
| 14 | PAK1 |  |  | 781 | 0.505 | 0.1200 | No |
| 15 | GADD45A |  |  | 867 | 0.480 | 0.1131 | No |
| 16 | RHOB |  |  | 881 | 0.476 | 0.1239 | No |
| 17 | CASP2 |  |  | 884 | 0.474 | 0.1374 | No |
| 18 | IER3 |  |  | 1010 | 0.442 | 0.1195 | No |
| 19 | CDKN1A |  |  | 1088 | 0.419 | 0.1128 | No |
| 20 | BGN |  |  | 1105 | 0.416 | 0.1211 | No |
| 21 | IRF1 |  |  | 1173 | 0.403 | 0.1163 | No |
| 22 | DAP3 |  |  | 1305 | 0.377 | 0.0950 | No |
| 23 | SLC20A1 |  |  | 1400 | 0.357 | 0.0823 | No |
| 24 | ETF1 |  |  | 1507 | 0.339 | 0.0661 | No |
| 25 | BIRC3 |  |  | 1626 | 0.320 | 0.0463 | No |
| 26 | JUN |  |  | 1725 | 0.307 | 0.0311 | No |
| 27 | CCND1 |  |  | 1753 | 0.304 | 0.0334 | No |
| 28 | TXNIP |  |  | 1918 | 0.283 | 0.0011 | No |
| 29 | EMP1 |  |  | 1987 | 0.274 | -0.0076 | No |
| 30 | CASP1 |  |  | 1994 | 0.273 | -0.0011 | No |
| 31 | DFFA |  |  | 2002 | 0.272 | 0.0052 | No |
| 32 | PEA15 |  |  | 2209 | 0.251 | -0.0383 | No |
| 33 | XIAP |  |  | 2355 | -0.265 | -0.0664 | No |
| 34 | HGF |  |  | 2596 | -0.300 | -0.1170 | No |
| 35 | IGF2R |  |  | 2736 | -0.324 | -0.1418 | No |
| 36 | MMP2 |  |  | 2762 | -0.328 | -0.1383 | No |
| 37 | CFLAR |  |  | 2781 | -0.332 | -0.1330 | No |
| 38 | ADD1 |  |  | 2886 | -0.353 | -0.1483 | No |
| 39 | SQSTM1 |  |  | 3013 | -0.378 | -0.1684 | No |
| 40 | DPYD |  |  | 3074 | -0.391 | -0.1717 | No |
| 41 | CREBBP |  |  | 3329 | -0.466 | -0.2208 | No |
| 42 | GSN |  |  | 3487 | -0.537 | -0.2439 | No |
| 43 | ERBB3 |  |  | 3561 | -0.578 | -0.2449 | Yes |
| 44 | IFITM3 |  |  | 3578 | -0.589 | -0.2315 | Yes |
| 45 | SAT1 |  |  | 3659 | -0.653 | -0.2321 | Yes |
| 46 | TIMP2 |  |  | 3705 | -0.691 | -0.2229 | Yes |
| 47 | CTNNB1 |  |  | 3753 | -0.731 | -0.2130 | Yes |
| 48 | PLAT |  |  | 3766 | -0.752 | -0.1938 | Yes |
| 49 | TGFB2 |  |  | 3774 | -0.766 | -0.1730 | Yes |
| 50 | SATB1 |  |  | 3776 | -0.767 | -0.1507 | Yes |
| 51 | FEZ1 |  |  | 3806 | -0.794 | -0.1345 | Yes |
| 52 | CDKN1B |  |  | 3812 | -0.800 | -0.1121 | Yes |
| 53 | GPX4 |  |  | 3915 | -0.986 | -0.1083 | Yes |
| 54 | BTG2 |  |  | 3917 | -0.999 | -0.0792 | Yes |
| 55 | CTH |  |  | 3970 | -1.112 | -0.0593 | Yes |
| 56 | APP |  |  | 3982 | -1.164 | -0.0277 | Yes |
| 57 | CLU |  |  | 4069 | -1.908 | 0.0072 | Yes |
Table: GSEA details [plain text format]

  

Fig 2: HALLMARK\_APOPTOSIS: Random ES distribution      
 Gene set null distribution of ES for **HALLMARK\_APOPTOSIS**
